# Digital Light Processing of Freeze-cast Ceramic Layers for Macroporous Calcium Phosphate Scaffolds with Tailored Microporous Frameworks

**DOI:** 10.3390/ma12182893

**Published:** 2019-09-07

**Authors:** Jong-Woo Kim, Jung-Bin Lee, Young-Hag Koh, Hyoun-Ee Kim

**Affiliations:** 1 School of Biomedical Engineering, Korea University, Seoul 02841, Korea (J.-W.K.) (J.-B.L.); 2 Department of Materials Science and Engineering, Seoul National University, Seoul 08826, Korea

**Keywords:** digital light processing, porogen, freeze-casting, porous ceramic scaffolds, hierarchical pores

## Abstract

The objective of the present study is to demonstrate the versatility of the digital light processing (DLP) technique particularly when using a freeze-cast ceramic layer as the feedstock, which can manufacture porous calcium phosphate (CaP) scaffolds with arbitrarily designed macroporous structures with tailored microporous frameworks specially designed for bone scaffold applications. For this goal, we employed camphene-camphor as the freezing vehicle and porogen for the preparation of photocurable CaP suspensions containing diurethane dimethacrylate (UDMA) monomers. After freeze-casting, the CaP suspensions could be solidified at controlled temperatures (~33–38 °C) and then be photopolymerized by DLP. All produced CaP scaffolds fairly resembled the designed macroporous structures (the gyroid structure with two interpenetrating macropore networks). In addition, numerous micropores were created in the CaP filaments, while the microporosity increased with increasing the camphene-camphor amount from 40 vol % to 60 vol %. As a consequence, compressive strength and modulus of hierarchically porous CaP scaffolds decreased due to an increase in overall porosity. However, reasonable mechanical properties could be obtained at high porosities owing to the CaP frameworks constructed in a periodic manner. In addition, excellent water penetration capability, biocompatibility, and apatite-forming ability were obtained, which were attributed to the microporous CaP frameworks with good pore interconnectivity and large surface area.

## 1. Introduction

Calcium phosphate (CaP) ceramics have been extensively used as bioactive materials for bone substitute applications [1,2,3]. In particular, porous calcium phosphate (CaP) scaffolds can facilitate bone ingrowth into pores, thus accelerating bone regeneration when implanted into bone defect areas [4]. For successful clinical uses, these porous materials should possess proper mechanical strengths that can withstand applied loads during bone regeneration. However, the higher porosity required for faster bone regeneration inevitably causes a severe reduction in mechanical strength [5]. This tradeoff relationship is one of the most critical hurdles for the designing of new types of porous ceramic scaffolds [6]. Thus, considerable attention has been paid to mimic the hierarchically porous structure of bones [5,6,7,8,9] in order to offer large surface areas and favorable paths for angiogenesis for new bone formation while providing reasonable mechanical properties [10,11,12,13,14]. 

In recent years, additive manufacturing (AM) techniques have opened new avenues in the manufacturing of porous scaffolds with advanced functions, since they can construct arbitrarily designed external shapes and internal porous structures, thus providing mechanical and biological functions tailored for the tissue regeneration of specific tissues and organs [15,16,17,18]. In addition, porous ceramic scaffolds can have unprecedentedly high mechanical strengths at high porosities owing to their tailored porous structures, which are hardly obtainable using traditional manufacturing techniques [19,20,21,22,23,24,25]. Consequently, these porous ceramic scaffolds would widen their practical uses for bone regeneration. However, only a few attempts have recently utilized AM techniques to manufacture hierarchically porous ceramic structures, presumably due to the difficulty in the preparation of ceramic-based feedstocks that are stable during and/or after AM processes [26]. For this goal, porogens are generally added to ceramic feedstocks, which can then be removed through various routes, thus leaving micropores in ceramic frameworks. For example, solid polymer microspheres [11,14,27] and air bubbles [28,29,30] have been directly incorporated into ceramic suspensions for direct-ink-writing (DIW). In addition, solidified crystals formed in freeze-cast ceramic feedstocks, which can be removed by sublimation, have been used for ceramic/camphene-based 3D extrusion process [31,32,33]. However, the macrostructures of hierarchical porous ceramic structures are limited due to the simple geometry of filaments used for extrusion-based AM techniques. To overcome this limitation, our group recently proposed photocurable ceramic slurries containing camphene-camphor as the porogen for digital light processing (DLP) [34], which would provide opportunities to manufacture complicated macrostructures with a high degree of design freedom.

**Figure 1:**
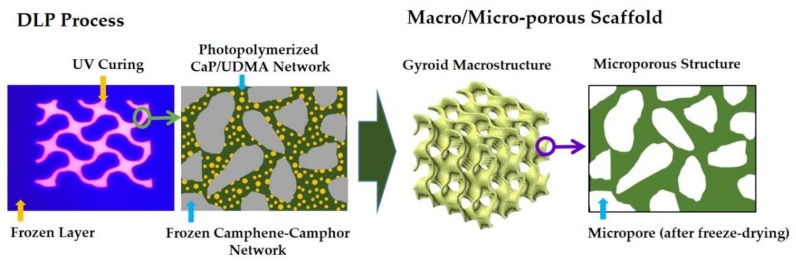
Schematic diagram showing the manufacturing of a macroporous calcium phosphate (CaP) scaffold with a gyroid macrostructure comprised of microporous frameworks using our recently developed DLP technique. Micropores can be created by removing the camphene-camphor network through freeze-drying.

In this study, we manufactured hierarchically porous CaP scaffolds with a gyroid macroporous structure comprised of tailored microporous frameworks using our newly developed DLP process [34] and characterized their mechanical properties and biological functions for bone scaffold applications. Our DLP process can selectively photopolymerize the thin layers of a freeze-cast CaP feedstock composed of three-dimensionally interconnected CaP/diurethane dimethacrylate (UDMA) monomers and solidified camphene-camphor networks (Figure 1). Micropores can be then created after the removal of the camphene-camphor networks through freeze-drying. In particular, the effect of the microporosity of the CaP frameworks on the overall porosities, mechanical properties, and in vitro biological properties (i.e., water penetration ability, biocompatibility, and apatite-forming ability) of the hierarchically porous CaP scaffolds was examined. 

## 2. Materials and Methods

### 2.1. Compositions of CaP Suspensions

Table 1 summarizes the compositions of a CaP suspension containing a camphene-camphor as the freezing vehicle and pore-forming agent for DLP process, which were determined based on our previous work [34]. Unless otherwise specified, all reagents were purchased from Sigma-Aldrich (St. Louis, MO, USA). Calcium phosphate (CaP) powder (Sun Medical, Gyeonggi-do, Korea) was used as the ceramic material owing to its high bioactivity and bone regeneration ability [2,3], and it is comprised of hydroxyapatite (HA, Ca_10_(PO_4_)_6_(OH)_2_) and β-tricalcium phosphate (β-TCP, β-Ca_3_(PO_4_)_2_) with a weight ratio of 60:40 (manufacturer’s data report). The particle size of the as-received CaP powder, measured using a laser diffraction particle size analyzer (Cilas 1090, Orleans, France), was in the range of 0.6–2.6 μm. A camphene and camphor mixture with a weight ratio of 2:1 was used as the freezing vehicle and porogen [34,35,36]. We employed diurethane dimethacrylate (UDMA) as the photopolymerizable monomer by considering its viscosity and mixing behavior with the camphene-camphor mixture. The viscosity of the UDMA, measured using a cone/plate viscometer (Discovery HR-2, TA instrument, New Castle, DE, USA), was 14.8 Pa⋅s. Phenylbis(2,4,6-trimethyl benzoyl)phosphine oxide was used as the photoinitiator, and oligomeric polyester dispersant (KD4, Croda, Everberg, Belgium) was used to improve the dispersion of the CaP powders.

### 2.2. Preparation of CaP Suspensions

Three types of CaP suspensions with a range of camphene-camphor amounts (40 vol %, 50 vol %, and 60 vol %) were used to control the microporosity. The predetermined amounts of the camphene/camphor alloy were added to CaP/monomer mixtures with 3 wt % of the dispersant and then ball-milled at 70 °C for 2 h. After, the prepared CaP suspensions were ball-milled with 2 wt % of the photoinitiator for 1 h at 70 °C for the DLP process.

### 2.3. Manufacturing of Hierarchically Porous CaP Scaffolds

Hierarchically porous CaP scaffolds with a gyroid macrostructure comprised of microporous frameworks were manufactured using the custom-made DLP machine (Figure 1). Unlike in a conventional DLP process using flowable ceramic suspensions, a warm ceramic suspension was deposited onto a previously photopolymerized layer and then solidified at controlled temperatures, as summarized in Table 2. The UV power and exposure time were also controlled to effectively induce the photopolymerization of the CaP layers.

To manufacture hierarchically porous CaP scaffolds with a gyroid macrostructure, CaP layers with a thickness of ~220 μm were photopolymerized for a constant exposure time of 20 s. This DLP process was repeated to create a gyroid macrostructure with pore sizes of 1 mm × 1.5 mm. The frozen camphene-camphor dendrites in the as-built CaP scaffolds were removed by freeze-drying for 24 h and then heat-treated for debinding and sintering at 1250 °C for 3 h [34].

### 2.4. Characterization of Macrostructure and Microporous Structures

Three types of hierarchically porous CaP scaffolds were manufactured with a range of camphene-camphor contents (40 vol %, 50 vol %, and 60 vol %) and their macro/micro-porous structures, mechanical properties, and in vitro biological functions were characterized using various analysis tools. The 3D macrostructures of hierarchically porous CaP scaffolds were characterized by optical microscopy (Samsung, Gyeonggi-do, Korea), The internal 3D macroporous structure was also examined by micro-computed tomography (μ-CT; Skyscan 1173 X-ray Micro-Tomography System, Skyscan, Kontich, Belgium). Field emission scanning electron microscopy (FE-SEM; JSM-6701F, JEOL Techniques, Tokyo, Japan) was utilized to examine the macroporous and microporous structures of the hierarchically porous CaP scaffolds. X-ray diffraction (XRD; M18XHF-SRA, MacScience Co., Yokohama, Japan) and energy dispersive X-ray spectrometry (EDS, Oxford Instruments, High Wycombe, UK) were used to characterize the crystalline phases and chemical compositions, respectively.

The overall porosities of the hierarchically porous CaP scaffolds were computed by comparing the measured density with theoretical density (*ρ*_s_ = 3.14 g/cm^3^ [2,3]). The microporosity of the CaP framework was also calculated using Archimedes’ principle. Mercury porosimetry (Micro Active AutoPore V 9600, Micromeritics Instrument Co., Norcross, GA, USA) was used to characterize the size distributions of the micropores formed in the CaP frameworks.

### 2.5. Measurement of Compressive Strengths and Modulus

The mechanical properties of the hierarchically porous CaP scaffolds manufactured with a range of camphene-camphor amounts (40–60 vol %) were characterized by compressive strength tests. Porous scaffolds (~7.9 × 7.9 × 7.3 mm) were compressed using a screw-driven load frame (OTU-05D, Oriental TM Corp., Gyeonggi-do, Korea). For these tests, a constant crosshead speed of 1 mm/min was used to monitor the fracture behaviors of the scaffolds. The compressive strengths and modulus were computed from the stress–strain curves recorded during the tests.

### 2.6. Evaluation of Water Penetration Ability

The water penetration ability of the hierarchically porous CaP scaffolds manufactured with a range of camphene-camphor amounts (40–60 vol %) was roughly determined by immersing their bottom in water; water could migrate from the bottom to the top through the microporous CaP frameworks. For clear observation, red dye was added to the water. After various times of immersion, the optical images of the top surfaces of the scaffolds were taken. 

### 2.7. Evaluation of In Vitro Cytocompatibility

The in vitro cytocompatibility of the hierarchically porous CaP scaffolds manufactured with a range of camphene-camphor amounts (40–60 vol %) was evaluated. For bone scaffold applications, a pre-osteoblast cell line (MC3T3-E1; ATCC, CRL-2593, Rockville, MD, USA) was used [37]. To examine the effect of microporosity on the cell attachment and proliferation, samples with dimensions of ~10 × 10 × 1 mm were tested. In addition, a tissue culture plate (Falcon™ Standard Tissue Culture Dishes, USA) was used as the positive control. The samples were sterilized with 70% ethanol for 12 h under ultraviolet (UV) irradiation.

Subsequently, the preincubated cells were seeded on the samples at a density of 2 × 10^4^ cells/mL. The MC3T3-E1 cells were cultured in a humidified incubator in an atmosphere containing 5% CO_2_ at 37 °C. As the culturing medium, a minimum essential medium (α-MEM: Welgene Co., Ltd., Seoul, Korea) supplemented with 10% fetal bovine serum (FBS), 1% penicillin-streptomycin, 10 mM β-glycerophosphate (Sigma), and 10 µg/mL ascorbic acid was used. 

After 24 h of cell culturing, the morphologies of the MC3T3-E1 cells adhered to and spread on the microporous CaP frameworks were observed using confocal laser scanning microscopy (CLSM; C1plus, Nikon, Tokyo, Japan). For these CLSM observations, the cultured cells were fixed with 4% paraformaldehyde, washed in PBS (phosphate buffered saline), and permeabilized with 0.1% Trion X-100 in PBS for 5 min. Subsequently, actin and cell nuclei were stained with fluorescent phalloidin (Alexa Fluor 555 phalloidin, Invitrogen, Waltham, MA, USA) and 4′,6-diamidino-2-phenylindole (DAPI; ProLong Gold antifade reagent with DAPI, Invitrogen, USA), respectively. The stained substrates were placed on a cover slide, and the cell morphologies were observed.

The cell proliferation rate was examined using a methoxyphenyl tetrazolium salt (MTS) assay with 3-(4, 5-dimethylthiazol-2-yl)-5-(3-carboxymethoxyphenyl)-2-(4-sulfophenyl)-2H-tetrazolium (MTS, Promega, Madison, WI, USA) for mitochondrial reduction. After three days of cell culturing, the quantity of the formazan product was measured by light absorbance at 490 nm using a microplate reader, which can be used to estimate the number of living cells in the culture. Five samples were tested for each condition. 

### 2.8. Evaluation of In Vitro Apatite-Forming Ability

The in vitro apatite-forming ability of the hierarchically porous CaP scaffolds manufactured with a range of camphene-camphor contents (40–60 vol %) was evaluated. The porous scaffolds were soaked in the simulated body fluid (SBF) solution at a controlled temperature of 37 °C for 6 h and 24 h [38]. The morphologies and microstructures of apatite crystals precipitated on the microporous CaP frameworks was characterized by FE-SEM and EDS. 

### 2.9. Statistical Analysis

Five samples were tested for each condition. Experimental data were expressed as mean ± standard deviation. Statistical analysis was performed using a one-way analysis of variance (ANOVA) with Tukey post-hoc comparison. A *p* value < 0.05 (*) was considered statistically significant.

## 3. Results and Discussion

### 3.1. 3D Macrostructures of Hierarchically Porous CaP Scaffolds

**Figure 2:**
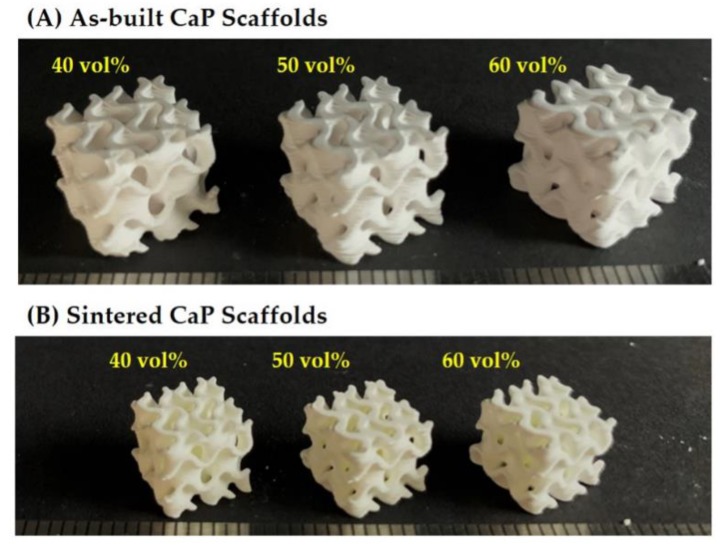
Optical images of (**A**) the as-built and (**B**) sintered CaP scaffolds with hierarchically porous structures manufactured with a range of camphene-camphor amounts (40 vol %, 50 vol %, and 60 vol %; scale = 1 mm).

Hierarchically porous CaP scaffolds comprised of an arbitrarily designed gyroid macrostructure with microporous CaP frameworks were successfully manufactured using a camphene-camphor mixture as the freezing vehicle and porogen for the DLP process [34]. In particular, we employed a gyroid structure for the construction of a macroporous structure. Since it can mimic unique structures found in nature, the gyroid structure provides opportunities for designing bone scaffolds with unprecedented functions [39]. In order to tune the microporosity and resulting overall porosity of hierarchically porous CaP scaffolds, the amount of camphene-camphor used as the porogen was controlled. Regardless of the camphene-camphor amounts (40–60 vol %), all produced CaP scaffolds showed tightly controlled macrostructures comprised of two interpenetrating macropore networks, as shown in Figure 2A. This finding indicates that the presence of solid camphene-camphor dendrites in a frozen layer would not severely interfere the photopolymerization of CaP suspensions, and thus designed macroporous structures can be successfully achieved. Furthermore, no noticeable defects (e.g., cracking and distortion) were observed for all of the CaP scaffolds after 1250 °C for 3 h, as shown in Figure 2B.

### 3.2. Internal Macroporous Structures

**Figure 3:**
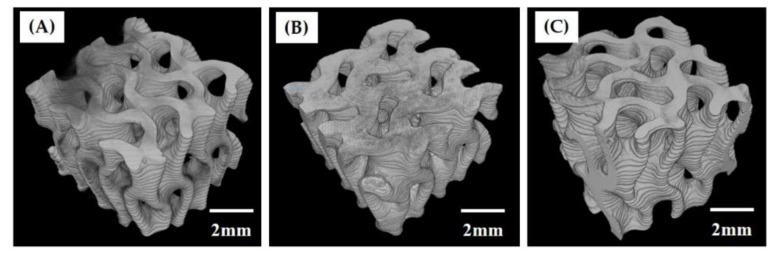
Representative micro-computed tomography (μ-CT) images of the hierarchically porous CaP scaffolds manufactured using a range of camphene-camphor amounts: (**A**) 40 vol %, (**B**) 50 vol %, and (**C**) 60 vol %.

To examine the three-dimensional macroporous structures of the hierarchically porous CaP scaffolds, the micro-CT analysis technique was utilized. Figure 3A–C shows representative μ-CT images of the hierarchically porous CaP scaffolds manufactured using a range of camphene-camphor amounts (40–60 vol %). All produced scaffolds revealed uniform macroporous structures comprised of gyroid unit cells. More specifically, two different types of CaP frameworks were constructed, alternately normal and parallel to the building direction. It should be noted that the macropores were perfectly interconnected, which is one of the most important prerequisites of porous ceramic scaffolds for favorable bone ingrowth [4,5].

### 3.3. Hierarchical Macrostructure and Microporous Structures

**Figure 4:**
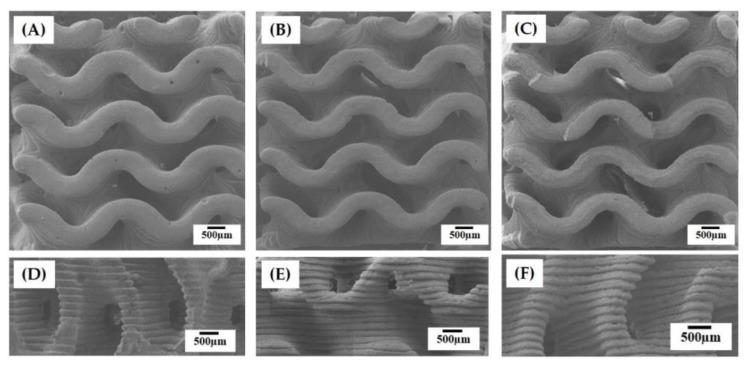
Representative field emission scanning electron microscopy (FE-SEM) images of the hierarchically porous CaP scaffolds manufactured using a range of camphene-camphor amounts of (**A**,**D**) 40 vol %, (**B**,**E**) 50 vol %, and (**C**,**F**) 60 vol %. The top (**A**–**C**) and bottom (**D**–**F**) images display the structures developed normal and parallel to the building direction.

Figure 4A–F shows representative FE-SEM images of the hierarchically porous scaffolds manufactured using a range of camphene-camphor amounts (40–60 vol %). All produced scaffolds displayed a characteristic of the gyroid structure, which has two interwoven macropore networks (i.e., macrochannels) (Figure 4A–C). In addition, smooth surfaces without noticeable features were formed for all the CaP frameworks. On the other hand, the side-view of the scaffolds showed grooved surfaces in the building direction that were created due to the UV scattering by CaP particles and resulting line broadening (Figure 4D–F). However, these rough, grooved surfaces would be expected to facilitate in the interaction between the material and cells [40]. 

**Figure 5:**
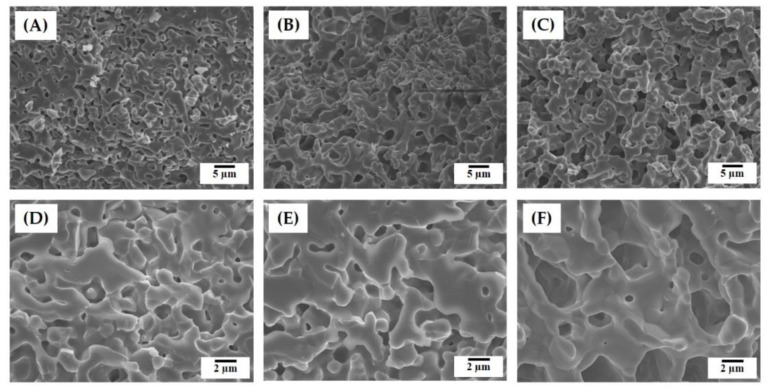
Representative FE-SEM images of the hierarchically porous CaP scaffolds manufactured using a range of camphene-camphor amounts showing their microporous structures: (**A**,**D**) 40 vol %, (**B**,**E**) 50 vol %, and (**C**,**F**) 60 vol %.

The microporous structures of the CaP frameworks obtained using a range of camphene-camphor amounts (40–60 vol %) are shown in Figure 5A–F. Regardless of the camphene-camphor amounts, a number of micropores were uniformly created in the CaP frameworks (Figure 5A–C). However, the microporosity increased remarkably with the increase of camphene-camphor amounts. In addition, the micropores became larger for higher camphene-camphor content (Figure 5D–F). Basically, micropores are formed through the sublimation of the camphene-camphor dendrites, and thus a higher camphene-camphor amount can provide higher microporosity with larger pore size [32,33,34]. Interestingly, the thin CaP walls were almost fully densified (Figure 5D–F).

### 3.4. Size Distributions of Micropores

**Figure 6:**
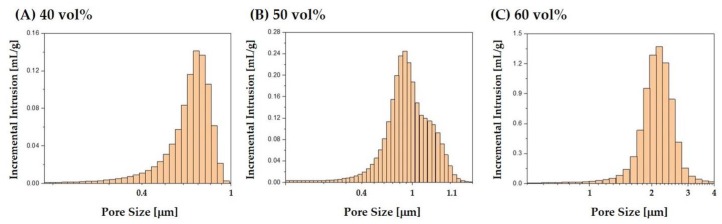
Size distributions of the micropores in the CaP frameworks obtained using a range of camphene-camphor amounts: (**A**) 40 vol %, (**B**) 50 vol %, and (**C**) 60 vol %.

The size distributions of the micropores formed in the CaP frameworks manufactured using a range of camphene-camphor amounts (40–60 vol %) are plotted in Figure 6A–C. All CaP frameworks displayed narrow pore size distributions, which is one of the strong benefits of the present approach using camphene-camphor as the porogen. The size ranges of 0.3–0.9 μm, 0.3–1.1 μm, and 1–4 μm were observed for the camphene-camphor amounts of 40 vol %, 50 vol %, and 60 vol %, respectively. However, higher freezing vehicle content resulted in large micropore size 

### 3.5. Overall Porosity and Microporosity

**Figure 7:**
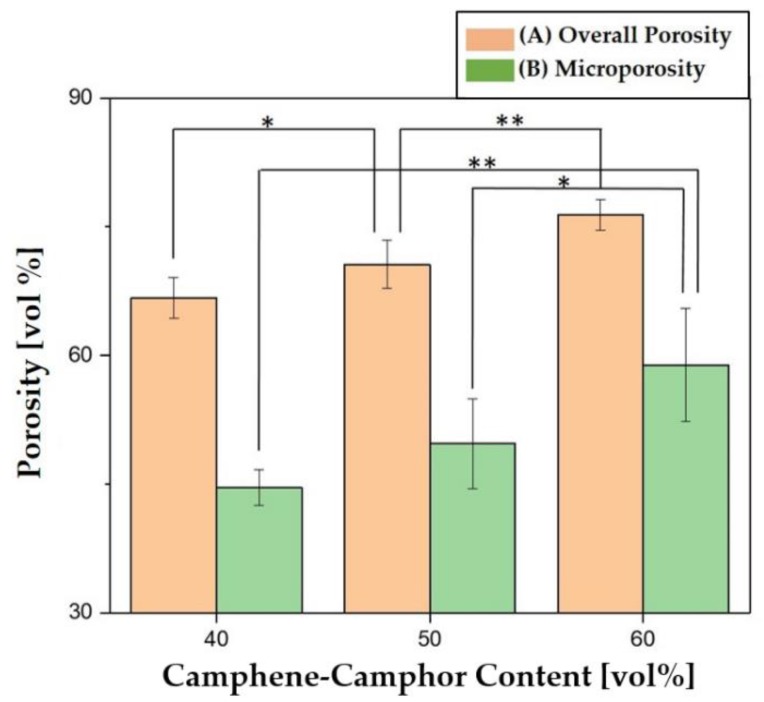
(**A**) Overall porosity and (**B**) microporosity of the hierarchically porous CaP scaffolds manufactured using a range of camphene-camphor amounts (40 vol %, 50 vol %, and 60 vol %). Five samples were tested for each condition (* *p* < 0.05 and ** *p* < 0.01).

The microporosity of the CaP frameworks and overall porosity of the hierarchically porous CaP scaffolds manufactured using a range of camphene-camphor amounts (40–60 vol %) were calculated using Archimedes’ principle and plotted in Figure 7A,B. The microporosity increased from 44.6 ± 2.1 vol % to 58.9 ± 6.6 vol % with the increase of camphene-camphor content from 40 vol % to 60 vol %, and, as a result, the overall porosity increased from 66.7 ± 2.4 vol % to 76.4 ± 1.8 vol %. This observation indicates that camphene-camphor can be effectively used as the porogen to tune the overall porosity of hierarchically porous CaP scaffolds.

### 3.6. Fracture Behavior, Compressive Strengths, and Modulus

**Figure 8:**
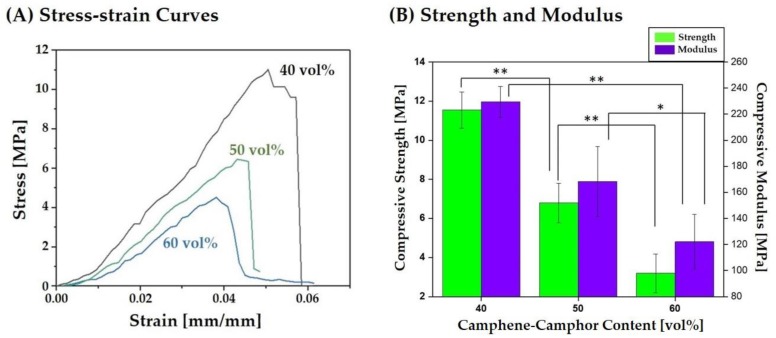
(**A**) Representative stress versus strain responses of the hierarchically porous CaP scaffolds manufactured using a range of camphene-camphor amounts (40 vol %, 50 vol %, and 60 vol %). (**B**) Compressive strengths and modulus as a function of the camphene-camphor amount. Five samples were tested for each condition (* *p* < 0.05 and ** *p* < 0.01).

Compressive strength tests were used to characterize the fracture behaviors and mechanical properties of the hierarchically porous CaP scaffolds manufactured using a range of camphene-camphor amounts (40–60 vol %). Figure 8A displays representative stress versus strain curves of three different types of hierarchically porous CaP scaffolds. All produced CaP scaffolds revealed similar fracture behaviors under compressive loads, in which stress increased almost linearly and then reached maximum value, followed by a rapid reduction without regimes of decreasing and increasing stress. These fracture behaviors indicate that the hierarchically porous CaP scaffolds can withstand applied compressive loads without the severe local collapse of the highly microporous CaP frameworks. The compressive strengths and modulus of the hierarchically porous CaP scaffolds computed from their stress–strain curves are plotted in Figure 8B. With the increase of camphene-camphor content from 40 vol % to 60 vol %, the compressive strength and modulus reduced from 11.5 ± 0.9 MPa to 3.2 ± 0.9 and from 229 ± 12 MPa to 122 ± 21 MPa, respectively. These reductions are primarily attributed to the increases in the microporosity and resulting overall porosity. However, a reasonable compressive strength of ~3.2 MPa could be achieved for the highest porosity of ~76 vol %, demonstrating their potential as the scaffolds for the repair and regeneration of cancellous bones [4,5,6].

### 3.7. Crystalline Phases

**Figure 9:**
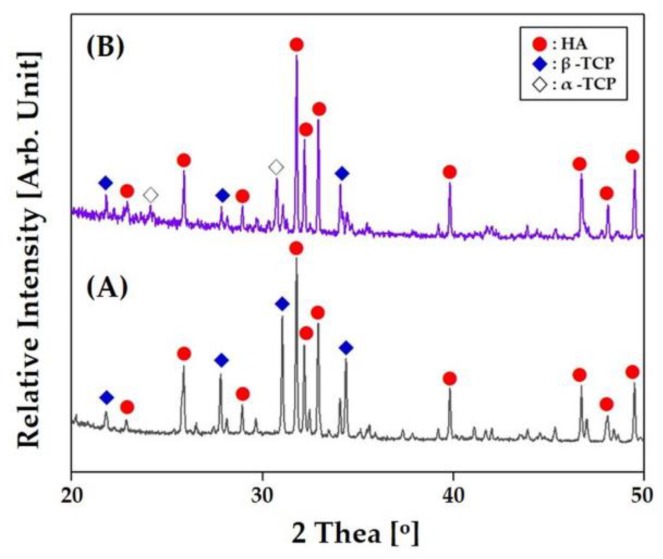
Representative X-ray diffraction (XRD) patterns of (**A**) the CaP powder and (**B**) hierarchically porous CaP scaffold after sintering at 1250 °C for 2 h.

The crystalline phases of the hierarchically porous CaP scaffold after sintering at 1250 °C for 3 h were characterized by XRD. As the reference, the CaP powder was also characterized. A number of strong peaks associated with hydroxyapatite (HA) and β- tricalcium phosphate (TCP) were observed [3]. However, compared to the as-received CaP powder (Figure 9A), additional peaks associated with α-TCP phase (JCPDS file No. 09–0348) were observed (Figure 9B). It is reasonable to suppose that this α-TCP phase is newly formed due to the phase transformation of β-TCP at 1250 °C for the sintering process [3,41]. These triphasic calcium phosphates have been demonstrated to have outstanding bioactivity in vitro and in vivo [3,41].

### 3.8. Water Penetration Capability

**Figure 10:**
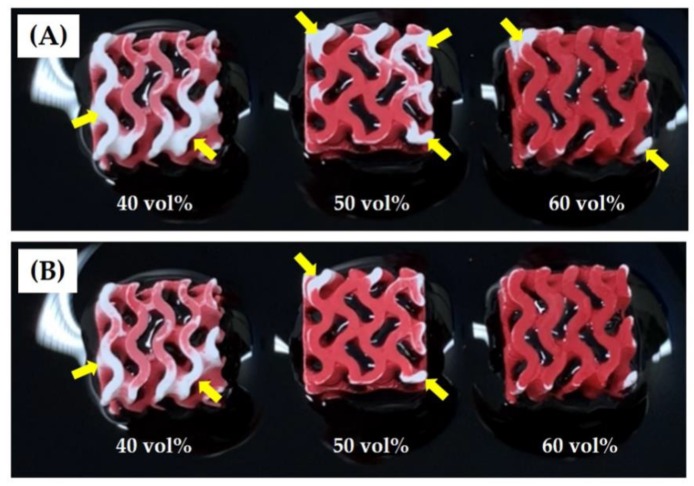
Optical image of the hierarchically porous CaP scaffolds manufactured using a range of camphene-camphor amounts (40 vol %, 50 vol %, and 60 vol %) after various immersion times of (**A**) 10 s and (**B**) 25 s. For clear observation, a red dye was added to the water. Yellow arrows indicate the regions without water penetration.

The water penetration capability of the hierarchically porous CaP scaffolds was roughly determined by monitoring their top surfaces after immersion in water. Figure 10A,B shows optical images of the top surfaces of the scaffolds manufactured with a range of camphene-camphor amounts (40–60 vol %) after 10 s and 25 s of immersion in water. Red dye was added to the water for clear observation. After 10 s of immersion, all of the scaffolds showed that the large areas of the CaP frameworks turned red due to the penetration of water from the bottom (Figure 10A). The yellow arrows indicate the regions without water penetration. However, the fraction of the red region increased remarkably with increasing the camphene-camphor amount, which is attributed to an increase in microporosity. After 25 s, the scaffold manufactured with the highest camphene-camphor content (60 vol %) showed that all of the CaP frameworks turned red, while some of the CaP frameworks still remained without water penetration for the scaffolds produced using lower camphene-camphor contents (40 vol % and 50 vol %) (Figure 10B). This observation indicates that the hierarchically porous CaP scaffolds composed of the microporous CaP frameworks with good pore interconnectivity would facilitate the transport of oxygen and nutrients for angiogenesis required for bone regeneration [9]. In addition, higher camphene-camphor content can provide higher microporosity with good pore interconnectivity, thus offering more favorable paths for mass transport.

### 3.9. In Vitro Cytocompatibility

**Figure 11:**
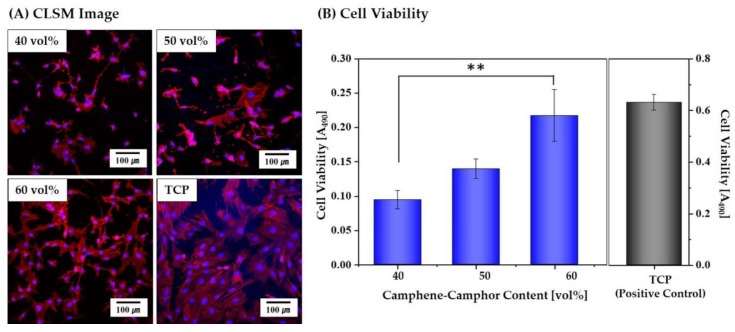
(**A**) Representative confocal laser scanning microscopy (CLSM) images of the MC3T3 cells adhered to and spread on the microporous CaP frameworks obtained using a range of camphene-camphor amounts (40 vol %, 50 vol %, and 60 vol %) after one day of cell culturing and (**B**) cell viabilities after three days of cell culturing. Tricalcium phosphate (TCP) was also tested as the positive control. Five samples were tested for each condition (* *p* < 0.05 and ** *p* < 0.01).

To examine the utility of the hierarchically porous CaP scaffolds with microporous frameworks for bone scaffold applications, their cytocompatibility was examined using in vitro cell tests. Figure 11A shows representative CLSM images of the MC3T3 cells adhered to and spread on the microporous CaP frameworks obtained using a range of camphene-camphor amounts (40–60 vol %). All scaffolds displayed similar cellular responses, i.e., many active cells spread across the surfaces of the microporous CaP frameworks. In addition, the cell viability after three days of cell culturing was measured by the MTS assay, which can be associated with the number of living cells. The microporous CaP framework obtained using the highest camphene-camphor content of 60 vol % showed significantly higher cell viability than that obtained using the lowest camphene-camphor content of 40 vol %, as shown in Figure 11B. It should be noted that the TCP used as the positive control had larger surface area than the microporous CaP frameworks, and thus higher cell viability was observed. These findings indicate that the cytocompatibility of the CaP can be preserved during the DLP process and after-heat-treatment and cell viability can be enhanced by creating microporous frameworks. 

### 3.10. In Vitro Apatite-forming Ability

**Figure 12:**
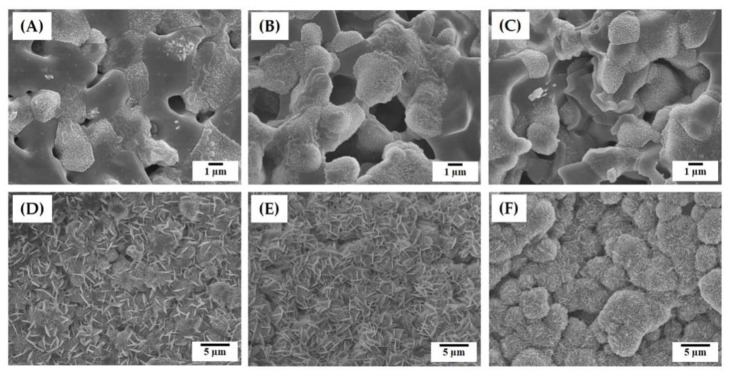
Representative FE-SEM images showing the surface morphologies of the microporous CaP frameworks obtained using a range of camphene-camphor amounts ((**A**,**D**) 40 vol %, (**B**,**E**) 50 vol %, and (**C**,**F**) 60 vol %) after immersion in the SBF solution for (**A**–**C**) 6 h and (**D**–**F**) 24 h.

The in vivo bioactivity of the hierarchically porous CaP scaffolds with microporous frameworks was roughly estimated by evaluating their in vitro apatite-forming ability in simulated body fluid (SBF) solution [38]. Basically, triphasic calcium phosphate, which is comprised of HA, β-TCP, and α-TCP, can offer outstanding apatite-forming ability, since α-TCP grains can dissolve preferentially in the SBF solution, thus releasing Ca^2+^ and PO_4_^3−^ ions favorable for apatite precipitation [36,37,38,39]. Figure 12A–F presents representative FE-SEM images showing the apatite crystals formed on the microporous CaP frameworks obtained using a range of camphene-camphor amounts (40–60 vol %) after 6 h and 24 h of immersion. After 6 h of immersion, the CaP framework obtained using the lowest camphene-camphor amount (40 vol %) showed the extensive dissolution of grains with an early stage of apatite precipitation (Figure 12A). On the contrary, the CaP framework obtained using higher camphene-camphor amounts (50 vol % and 60 vol %) showed vigorous precipitation of apatite crystals (Figure 12B,C). It should be noted that higher microporosity can provide a larger surface area and more favorable paths for grain dissolution and apatite precipitation. After 24 h of immersion, the CaP frameworks were fully covered with apatite crystals (Figure 12D–F). 

However, the morphologies of the apatite crystals was affected by the freezing vehicle content. The CaP framework obtained using camphene-camphor amounts of 40 vol % and 50 vol % showed a mixture of tiny flake-like and planar-like crystals (Figure 12D,E). On the other hand, the CaP framework obtained using the highest camphene-camphor amount of 60 vol % revealed granules comprised of tiny fake-like crystals, as is often the case with bioactive α-TCP and HA/TCP composites [41,42,43,44]. This excellent ability is mainly due to the achievement of high microporosity with good pore interconnectivity and large surface area for apatite precipitation. In addition, the formation of α-TCP phase after sintering at 1250 °C would be beneficial to apatite precipitation in the SBF solution.

## 4. Conclusions

Hierarchically porous CaP scaffolds with an arbitrarily designed macrostructure comprised of microporous frameworks having tailored microporosity were successfully manufactured by the DLP technique using a freeze-cast feedstock containing camphene-camphor as the porogen. Regardless of the camphene-camphor amounts (40–60 vol %), designed macroporous gyroid structures could be obtained for all CaP scaffolds revealed due to the use of the DLP process. In addition, CaP frameworks revealed a number of micropores that were created by removing the camphene-camphor dendrites through freeze-drying. With the increase of camphene-camphor content from 40 vol% to 60 vol %, the microporosity increased from ~44.6 vol % to ~58.9 vol %, and thus the overall porosity of the scaffolds increased from ~66.7 vol % to ~76.4 vol %. Consequently, mechanical properties decreased; however, a reasonable compressive strength (~3.2 MPa) was obtained even for the scaffold with a high porosity of ~76.4 vol %. No cytotoxicity was observed for all CaP scaffolds. Water penetration capability and in vivo apatite-forming ability increased with the increase of camphene-camphor content, which was due to an increase in microporosity. These findings indicate the great usefulness of hierarchically porous CaP scaffolds with microporous frameworks for bone scaffold applications.

## Figures and Tables

**Table 1:** Compositions of CaP ceramic suspensions used for the digital light processing (DLP) process.

Role	Ceramic Powder	Photocurable Monomer	Freezing Vehicle	Dispersant	Photoinitiator
Material	CaP	UDMA	2 Camphene + 1 Camphor	KD4	PPO
Amount (g)	47.6	15	18.14–40.8 ^(1)^	1.47 ^(2)^	0.3 ^(3)^

^(1)^ 18.2 g, 27.2 g, and 40.8 g for the camphene-camphor contents of 40 vol %, 50 vol %, and 60 vol %, respectively. ^(2)^ 3 wt % with respect to the CaP content. ^(3)^ 2 wt % with respect to the diurethane dimethacrylate (UDMA) content.

**Table 2:** Processing parameters used for the manufacturing of the hierarchically porous CaP scaffolds.

Freezing Conditions		Photopolymerization Conditions		
Temperature of CaP Slurry	Temperature of Building Platform	Layer Thickness	UV Power	Exposure Time
70 °C	~33–38 °C ^†^	~220 μm	60 W/m^2^	20 s

^†^ ~33 °C, 36 °C, and 38 °C for the CaP suspensions with camphene-camphor amounts of 40 vol %, 50 vol %, and 60 vol %, respectively.
